# Development of global health education at Johns Hopkins University School of Medicine: a student-driven initiative

**DOI:** 10.3402/meo.v20.28632

**Published:** 2015-07-27

**Authors:** Dane Moran, Jill Edwardson, Charles Nicholas Cuneo, Sean Tackett, James Aluri, Antony Kironji, Jacob Cox, Bryn Carroll, Erina Lie, Mariam Fofana, Robert C. Bollinger, Roy C. Ziegelstein, Chi C. G. Chen

**Affiliations:** Department of Obstetrics and Gynecology, Johns Hopkins University School of Medicine, Baltimore, MD, USA

**Keywords:** global health, leadership, program planning/curriculum development, innovative educational interventions, interprofessional education

## Abstract

Global health is increasingly present in the formal educational curricula of medical schools across North America. In 2008, students at Johns Hopkins University School of Medicine (JHUSOM) perceived a lack of structured global health education in the existing curriculum and began working with the administration to enhance global health learning opportunities, particularly in resource-poor settings. Key events in the development of global health education have included the introduction of a global health intersession mandatory for all first-year students; required pre-departure ethics training for students before all international electives; and the development of a clinical global health elective (Global Health Leadership Program, GHLP). The main challenges to improving global health education for medical students have included securing funding, obtaining institutional support, and developing an interprofessional program that benefits from the resources of the Schools of Medicine, Public Health, and Nursing. Strategies used included objectively demonstrating the need for and barriers to more structured global health experiences; obtaining guidance and modifying existing resources from other institutions and relevant educational websites; and harnessing institution-specific strengths including the large Johns Hopkins global research footprint and existing interprofessional collaborations across the three schools. The Johns Hopkins experience demonstrates that with a supportive administration, students can play an important and effective role in improving global health educational opportunities. The strategies we used may be informative for other students and educators looking to implement global health programs at their own institutions.

Interest in global health has increased substantially over time, as reflected by the number of medical students that undertake international rotations while in medical school (1). In 1984, the Graduation Questionnaire administered by the Association of American Medical Colleges (AAMC) found that 6% of students had an international experience during medical school; by 2014, that figure had increased to 29% (2). However, despite a definite increase in global health opportunities in U.S. medical schools, 36% of students in 2013 still felt that they received inadequate education in this area (3).

Participating students find global health opportunities to be very valuable. In addition to gaining knowledge and experiences specific to global health, students demonstrate improved physical diagnostic and clinical decision-making skills, often related to the experience of practicing in settings where access to imaging and laboratory services are limited. Students participating in global health electives report increased personal development and awareness of the social determinants of health (4). Furthermore, they are found to be more culturally competent and are more likely to practice in primary care settings and to serve multicultural and underserved populations (5, 6).

Since the University of Arizona established the first formalized global health curriculum in 1992, many other U.S. medical schools have sought to do the same (7–11). In 2011, 32 of 141 accredited MD-granting medical schools in the U.S. had structured global health programs, with 40% of these requiring an international clinical experience (12). The increased number of these programs reflects both an acknowledgement of student interest in the formal inclusion of global health into medical school curricula as well as its importance.

Developing a global health program can seem to be a daunting task for any institution, but especially as a medical student-driven initiative. Here, we present a timeline of key events and elaborate on the barriers to and solutions that were required for development of a global health curriculum at Johns Hopkins University School of Medicine (JHUSOM). Our aim is to share our experiences so that educators and students from other institutions may consider some of our strategies in the development of their global health programs.

## History of development

**Fig. 1:**
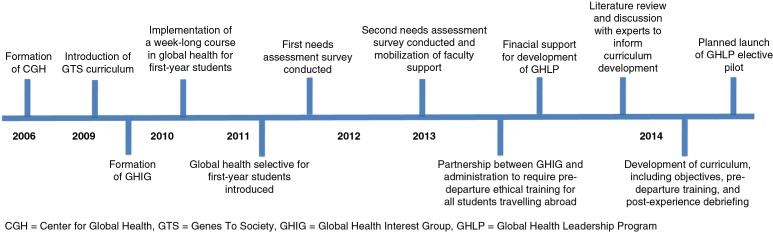
Overview of the timeline of events crucial for the development and implementation of a structured global health curriculum at Johns Hopkins.

A global health curriculum at JHUSOM has developed over the last decade (Fig. 1). A key first step was the creation of the Johns Hopkins Center for Global Health (CGH) in 2006, whose mission is to focus the global health expertise and resources at Johns Hopkins to effectively address and ameliorate the world's most pressing health issues. CGH promotes international research, policy development, and the development of leaders in global health both domestically and internationally. The center further functions to facilitate and coordinate international activities for students across the Schools of Medicine (SOM), Nursing (SON), and Public Health (SPH), which are conveniently located on the same campus (13). CGH provides international summer research opportunities for medical students between their first and second years and keeps a central repository of Johns Hopkins clinical and research activities abroad. While the advent of CGH made it easier for motivated students to identify opportunities for international research and clinical experiences, there was no formal program that provided oversight of the clinical setting, faculty, and expected level of medical student responsibility for these experiences. Importantly, without the development of a formalized, clinical global health elective, it was difficult for students to individually incorporate international clinical experiences into their education. According to the AAMC Graduation Questionnaire, approximately 30% of Johns Hopkins medical students undertake international experiences. Such experiences have traditionally been independently arranged by students and approved on an *ad hoc* basis by the Office of Student Affairs (OSA) at JHUSOM.

In 2009, JHUSOM implemented the new Genes to Society (GTS) curriculum, which included a series of intersession courses, ‘Topics in Interdisciplinary Medical Education’ (TIME) (14). These TIME courses occur throughout the 4-year curriculum, each lasting for approximately 1 week. Students seized upon this moment of curricular change and approached a faculty member at JHUSOM to request that the topic of global health be included as one of these TIME courses. After receiving administrative support, the TIME course on global health was developed and taught for the first time in spring 2010 to all medical students (15). Due to positive feedback from students regarding the course, the medical school administration incorporated it as a permanent and mandatory component of the curriculum, to be taught yearly to all first-year students. This exemplifies the positive changes that can be brought about by motivated students capitalizing on a time of curricular change in the setting of a supportive administration.

In 2009, a small group of interested medical students established the Global Health Interest Group (GHIG), which has played a pivotal role in partnering with faculty and administration to further advance global health education within the SOM (Table 1). The leaders of GHIG identified experienced faculty to serve as mentors for the interest group, in particular JHUSOM faculty in leadership positions at CGH. In 2011, GHIG members organized a global health selective to offer to interested first-year students. The selective includes three 2-hour sessions that are organized by medical students and are overseen by faculty as part of students’ required Foundations of Public Health pre-clinical course. Further, in 2012, GHIG successfully partnered with the administration to institute compulsory ethical training before any student travels abroad, which involves completing an online module that was developed by Johns Hopkins University's Berman Institute of Bioethics and Stanford University (16).

**Table 1:** Barriers and solutions to the adoption of a global health curriculum at Johns Hopkins School of Medicine

Barriers	Solutions
Multiple student and institutional interests	• Implement a Global Health Interest Group (GHIG) to facilitate student interest, coordinate global health-related activities, and develop a presence on campus • Objectively demonstrate student interest through a needs assessment survey • Administrative support and student empowerment
Funding to support a structured global health program	• Objectively demonstrate the interest for a structured program and the importance of financial support to student participation through a needs assessment survey • Obtain support from the institution to help with targeted fundraising • Apply for outside grants
Developing an innovative global health program	• Capitalize on institutional strengths, such as close links between SOM, SON and SPH and a large global research footprint • Review literature and web resources • Seek guidance from other universities with established programs
Coordinating the efforts of three Schools at Johns Hopkins	• Clear communication between SOM, SON, and SPH leadership • Use students to facilitate communication between faculty in different departments/schools that have global health expertise
Modifying an established curriculum	• Take advantage of moments of curricular change to advocate for a course in global health
Lack of a formalized international elective experience	• Survey students to objectively demonstrate desired qualities in a global health program • Implement and standardize pre-departure training

SOM: School of Medicine; SON: School of Nursing; SPH: School of Public Health.

In 2012, GHIG surveyed first- through third-year medical students to gauge interest in international clinical electives. The survey, with a response rate of 30% (108 total responses), revealed that a majority of students (94.4%) did not feel knowledgeable about available resources for identifying international electives despite abundant interest in international opportunities (99% of respondents). Students’ comments emphasized a desire for better-organized and structured global health opportunities. GHIG approached the OSA and CGH with these survey results and suggested the development of a formalized international clinical elective program. Important first steps were taken by the CGH leadership to identify possible sites for such a program, while the OSA provided input on the necessary elements for an elective to meet approval for credit. These meetings highlighted the importance of having Johns Hopkins faculty present at international sites where students train, of establishing a mutually advantageous partnership with international sites, and of financial and safety considerations for participating students. Although several sites meeting the criteria were identified at theses meetings, a formalized international clinical elective program was not established at the time.

To better advocate for dedicated funding for a structured international clinical elective, GHIG sought the support and assistance of additional faculty mentors and conducted a second, expanded survey with a 60.4% response rate (290 total responses) in the spring of 2013. Eighty percent of students who responded to this survey reported interest in undertaking an international clinical elective. Additionally, 90% of students listed financial assistance as either ‘very important’ or ‘important’ in influencing their decision to go abroad. In past years, students had been able to obtain only limited funding for electives abroad through CGH or through private endowments at the SOM that supported experiences in specific geographic regions.

Equipped with these data, the student leaders of GHIG collaborated with faculty mentors to draft a formal proposal to present to the Vice Dean for Education at JHUSOM and the Associate Dean for Student Affairs. Emphasis was placed on several areas, including interprofessional education, student safety, reciprocity with international partners, and the development of future leaders in global health. Particular attention was paid to ensuring that the new elective would satisfy the Liaison Committee on Medical Education (LCME) elements for elective rotations, including making certain that students receive appropriate supervision, teaching, and evaluation, and that the expected level of medical student responsibility is appropriate for the student's training. The proposal was accepted and a grant award was given to pilot an international clinical elective program. The willingness of the SOM administration to listen to the needs of the student body and to empower students to effect change were crucial elements that enabled the further development of a formalized international clinical elective program.

A taskforce comprised of GHIG members was then established to develop the elective, and a faculty program director was appointed. This taskforce consists of medical students, residents, fellows, and faculty from the SOM, SON, and SPH, and began monthly meetings in fall 2013. A thorough review of the literature and discussions with faculty from Johns Hopkins University (JHU) and other institutions helped develop an informed, comprehensive, and innovative clinical elective program, the Global Health Leadership Program (GHLP). In order to avoid ‘reinventing the wheel’ during this process, we sought advice and guidance from faculty and administrators at outside institutions with established global health clinical electives including Indiana University, University of Michigan, University of California at San Francisco, Harvard University, and University of Pennsylvania. We also used web-based resources from organizations such as the *Consortium of Universities for Global Health* to aid in the development of our curricular and didactic content.

As we wanted GHLP to be learner-centered, the specifics of the program, including curriculum and learning objectives, were developed primarily by medical students with guidance from faculty members. The faculty from SOM, SON, and SPH included experts in biomedical ethics, curricular/program development, medical education and simulation, leadership skills, global health, health management, women's health and urology, internal medicine, pediatrics, emergency medicine, surgery, and infectious diseases. The resulting curriculum incorporated the already required ethics modules from the Berman Institute of Bioethics along with global health didactic content from web-based resources, in-person sessions on health care delivery and management, leadership skills and simulation training, and a post-experience debriefing.

In particular, an emphasis was placed on developing a curriculum to facilitate the training and development of future leaders in global health. This effort led to the involvement of partners from organizations such as Jhpiego (an international non-profit organization affiliated with JHU) and the World Health Organization (WHO). Further, given our belief that healthcare is best taught and learned in an interprofessional environment and that interprofessional collaboration is necessary in clinical settings domestically and abroad, interprofessional education was emphasized in our program. This interprofessional approach enabled us to take advantage of existing resources and collaborations with SON and SPH and helped to facilitate the incorporation of interprofessional competencies into our program.

Additional activities of the GHLP taskforce included identification of potential elective sites and international faculty partners, grant writing to identify additional funding sources for the program, and the compilation of a pre-departure handbook. Notably, the taskforce also performed a needs assessment to identify what students felt was most important in a pre-departure program to prepare them for global health electives. Since one of the program's goals is to offer meaningful experiences to those interested in global health careers, we incorporated optional internship opportunities with organizations such as WHO and Jhpiego. Besides providing a structured global health elective for students, development of the GHLP also provided a structured framework for Johns Hopkins faculty to involve students in their global health activities and, in turn, to be involved in global health education.

## Elective site selection

Mindful selection of international sites and collaborative partners is an essential component of developing a successful international elective program. We used the following criteria to systematically evaluate potential international elective partners: 1) the presence and availability of Johns Hopkins faculty at the site; 2) the presence of local medical, nursing, and public health students; 3) the existence of an established relationship between the site and Johns Hopkins; 4) the ability and experience of the site to host international students; 5) the presence of appropriate clinical facilities and housing (on-site or off-site) facilities; and 6) the general safety and availability of transportation. It was also important to us that partners be in resource-poor settings to provide a diverse learning experience for our students and to promote exchange of ideas between the developed and developing world. The first partnership we have established is in Pune, India, and other partnerships will likely follow if the program is successful.

In this process of site selection, we capitalized on the long tradition of clinical, research, and educational collaboration between Johns Hopkins faculty and educators based internationally. Specifically, the presence of Johns Hopkins faculty located on site longitudinally was an important criterion for selecting elective sites because of their understanding of the educational mission and educational structure at both Johns Hopkins and the international site. Our on-site faculty member provides and/or facilitates the provision of on-the-ground clinical and/or research supervision and serves as a resource for the students during their time abroad. As we continue to develop this program, we hope to involve more of the international faculty at our partnering sites so that even without the presence of Johns Hopkins faculty, our program will be sustainable.

Additionally, it is important that our program is mutually beneficial for Johns Hopkins students and for students at our international partner sites. While abroad, our students work with local medical, nursing, and public health students in clinical and research settings. Specifically, our students along with their international counterparts are required to complete a clinical, public health, educational, or quality-improvement project on a topic identified or supported by local staff. GHLP is also working towards securing funding to allow students from our international partners in resource-poor settings to come to Hopkins for clinical and research electives.

## Summary

Over the last decade, students have worked with the medical school administration at JHUSOM to improve global health education. Key milestones included the incorporation of a global health intersession in the first-year SOM curriculum, requirement of ethics pre-departure training before any international electives, and the development of a clinical global health elective (GHLP). Challenges included securing funding, obtaining institutional support, and coordinating the involvement of faculty and expertise in the SOM, SON, and SPH. Our approach included objectively demonstrating the need for and barriers to more structured global health experiences with the use of surveys of the medical student body; presenting our proposal during times of administrative and/or curricular changes within the SOM; highlighting the importance of oversight of student supervision, teaching, and evaluation at international sites; obtaining guidance and modifying existing resources from other institutions and relevant educational websites; and importantly harnessing institutional specific strengths including the large Johns Hopkins global research footprint and existing interprofessional collaborations. The Johns Hopkins experience demonstrates the important and effective role that an organized and motivated group of students can play in improving global health educational opportunities with the support and guidance of faculty and administration.
